# Focus on Depression in Parkinson's Disease: A Delphi Consensus of Experts in Psychiatry, Neurology, and Geriatrics

**DOI:** 10.1155/2021/6621991

**Published:** 2021-02-08

**Authors:** Luis Agüera-Ortiz, Rocío García-Ramos, Francisco J. Grandas Pérez, Jorge López-Álvarez, José Manuel Montes Rodríguez, F. Javier Olazarán Rodríguez, Javier Olivera Pueyo, Carmelo Pelegrín Valero, Jesús Porta-Etessam

**Affiliations:** ^1^Servicio de Psiquiatría, Instituto de Investigación i+12, Hospital Universitario 12 de Octubre, Madrid, Spain; ^2^Centro de Investigación Biomédica en Red de Salud Mental (CIBERSAM), Madrid, Spain; ^3^Movement Disorders Unit, Servicio de Neurología, Hospital Clínico San Carlos, Complutense University, Madrid, Spain; ^4^Movement Disorders Unit, Hospital General Universitario Gregorio Marañón, Madrid, Spain; ^5^Service of Psychiatry, University Hospital Ramón y Cajal. CIBERSAM, IRYCIS. University of Alcalá, Madrid, Spain; ^6^Service of Neurology, HGU Gregorio Marañón, Madrid, Spain; ^7^Memory Disorders Unit, HM Hospitales, Madrid, Spain; ^8^Service of Psychiatry, Hospital Universitario “San Jorge”, Huesca, Spain; ^9^Neurological Rehabilitation Unit, Clínica Ubarmin, Pamplona, Navarra, Spain; ^10^Service of Neurology, Instituto de Neurociencias, Hospital Clínico San Carlos, San Carlos, IdISSC, Madrid, Spain

## Abstract

Major and minor forms of depression are significant contributors to Parkinson's disease morbidity and caregiver burden, affecting up to 50% of these patients. Nonetheless, symptoms of depression are still underrecognized and undertreated in this context due to scarcity of evidence and, consequently, consistent clinical guideline recommendations. Here, we carried out a prospective, multicentre, 2-round modified Delphi survey with 49 questions about the aetiopathological mechanisms of depression in Parkinson's disease (10), clinical features and connections with motor and nonmotor symptoms (10), diagnostic criteria (5), and therapeutic options (24). Items were assessed by a panel of 37 experts (neurologists, psychiatrists, and a geriatrist), and consensus was achieved in 81.6% of them. Depressive symptoms, enhanced by multiple patient circumstances, were considered Parkinson's disease risk factors but not clinical indicators of motor symptom and disease progression. These patients should be systematically screened for depression while ruling out both anhedonia and apathy symptoms as they are not necessarily linked to it. Clinical scales (mainly the Geriatric Depression Scale GDS-15) can help establishing the diagnosis of depression, the symptoms of which will require treatment regardless of severity. Efficacious and well-tolerated pharmacological options for Parkinson's comorbid depression were selective serotonin reuptake inhibitors (especially sertraline), dual-action serotonin and norepinephrine reuptake inhibitors (venlafaxine, desvenlafaxine, and duloxetine), multimodal (vortioxetine, bupropion, mirtazapine, and tianeptine), and anti-Parkinsonian dopamine agonists (pramipexole, ropinirole, and rotigotine). Tricyclic antidepressants and combining type B monoamine oxidase inhibitors with serotonergic drugs have serious side effects in these patients and therefore should not be prescribed. Electroconvulsive therapy was indicated for severe and drug-refractory cases. Cognitive behavioural therapy was recommended in cases of mild depression. Results presented here are useful diagnostic and patient management guidance for other physicians and important considerations to improve future drug trial design.

## 1. Introduction

Neuropsychiatric disorders (and nonmotor symptoms more widely) such as depression very often accompany core motor impairments of Parkinson's disease (PD) [1]. More and more, they are perceived as significant contributors to PD morbidity and caregiver burden as they have a major impact on patient's function, quality of life, and long-term outcomes. In fact, it has been suggested that PD should be classified as a neuropsychiatric disease itself rather than a movement disorder [2].

Up to half of PD patients suffer from major or minor forms of depression at a given time during the disease course, and although they have been widely studied in this context, they remain both underrecognized and undertreated [2, 3]. Standard diagnostic criteria for depression in PD have not been established, perhaps due to an existing overlap between motor and depression symptoms such as (fatigue, insomnia, and psychomotor slowing) as well as the co-occurrence of other nonmotor symptoms such as (anxiety and apathy) [4]. Similarly, therapeutic interventions for depression in the context of PD are neither definitive nor specific. Controlled pharmacological studies and clinical practice reports on antidepressants are still scarce and encompass important limitations such as small sample size, trial duration, and heterogeneity in the assessment and diagnosis of depression [5, 6]. Moreover, information on the management of depression and PD comorbid patients is usually extrapolated from depression, geriatrics, or PD clinical practice guidelines (CPGs), which leads to an increased variability of habits and disagreement among physicians [5, 7].

Hence, the aim of this study is to help homogenize diagnostic and treatment approaches to offer a higher quality healthcare assistance to PD patients with depression. To do it, a 2-round, Delphi questionnaire was used to gather psychiatry, neurology, and geriatrics experts' opinions and preferences based on their expertise along with current scientific evidence.

## 2. Materials and Methods

### 2.1. Study Design

This study uses the modified Delphi method [8–10] as in Agüera-Ortiz et al. and Luquin et al. [11, 12]. A two-round closed-ended questionnaire included 49 items revisiting some fundamental clinical aspects of depression in PD: aetiology and risk factors (10), clinical presentation (10), diagnosis (5), and treatment (24). In the second round, participants were presented with the results and comments from the first one. This way, items for which consensus had not been reached could be reassessed. The survey was available on a specifically designed website.

### 2.2. Participants

The Scientific Committee was composed of 8 expert physicians in the neurodegenerative diseases field (4 psychiatrists and 4 neurologists) from different Spanish centres. After careful review of the literature, they prepared the questionnaire and validated the study design and protocol. The committee was also in charge of adding the first-round statistical analysis and feedback to the online platform for subsequent reconsideration.

A panel of 37 physicians who routinely assist PD patients (19 neurologists, 17 psychiatrists, and 1 geriatrist) participated in both rounds of the study. They were selected by the committee following a snowball sampling procedure [13]. All of them bring more than 10 years of clinical practice as well as an established and verifiable academic and research career.

### 2.3. Delphi Methodology

Participants anonymously responded to the questionnaire using a single ordinal 9-point Likert scale: 1–3 (disagreement with the statement; the lower the score, the higher the level of disagreement), 4–6 (neither agreement nor disagreement; a score of 4 is closer to disagreement), and 7–9 (agreement with the statement; the higher the score, the higher the level of agreement). RAND/UCLA criteria was used, for which consensus is reached when less than one-third of participants rates the item outside the 3-point region (1–3, 4–6, and 7–9) containing the median [8, 10, 14]. Median value determined the nature of the consensus (≤3, disagreement; ≥7, agreement). Items were reanalysed in the second round if the median was between 4 and 6 or if one-third or more of the votes fell outside the 3-point region containing it.

### 2.4. Statistical Analysis

Results were interpreted based on the mean and the confidence interval for the average at a 95% confidence level for each item. The higher (closer to 9) or lower (closer to 1) the average, the stronger the collective agreement or disagreement with the statement, respectively. A high unanimity of opinions was mirrored by a low confidence interval. Nonconsensus items were also defined by an interval containing the value 5 (neither agreement nor disagreement). Such items were accompanied by a descriptive reasoning (there was either heterogeneity of opinions or a lack of definitive individual criteria).

## 3. Results

The modified Delphi survey on depression in PD patients included a total number of 49 items, which were responded by a panel of experts on neuropsychiatric disorders of the elderly.

**Figure 1:**
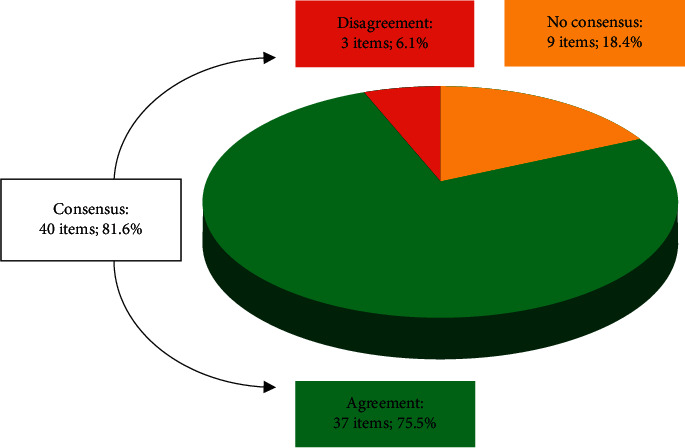
Overall results of the Delphi study: degree of consensus, statement agreement, and disagreement. *N* = 49 items.

Overall, consensus was achieved in most cases (40 statements; 81.6%), either in the first round (36; 73.5%) or in the second one (4; 8.2%; S5, S21, S22, and S25). The majority of them were accepted by the panellists (37; 75.5%). Only a small part of the questionnaire was left for future consideration as consensus could not be reached (9; 18.4%; S4, S8, S23, S30, S32, S37, S46, S47, and S49) (Figure 1). Agreed, disagreed, and nonconsensus items are given in detail in Tables 1–4 according to the following sections: aetiology and risk factors (consensus in 8 (80%) out of 10 statements; Table 1), clinical presentation (consensus in all 10 statements (100%); Table 2), diagnosis (consensus in 4 (80%) out of 5 statements; Table 3), and treatment of depression in PD patients (consensus in 18 (75%) out of 24 statements; Table 4).

## 4. Discussion

**Figure 2:**
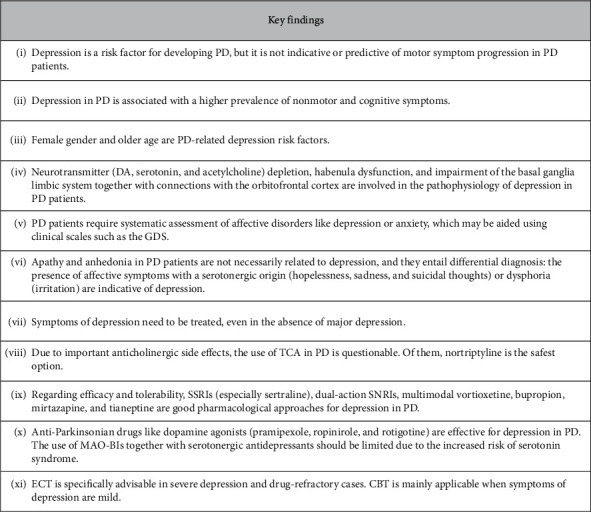
Highlights of the Delphi survey. PD, Parkinson's disease; DA, dopamine; GDS, Geriatric Depression Scale; TCA, tricyclic antidepressants; SSRI, selective serotonin reuptake inhibitors; SNRIs, serotonin and norepinephrine reuptake inhibitors; MAO-BIs, type B monoamine oxidase inhibitors; ECT, electroconvulsive therapy; CBT, cognitive behavioural therapy.

Participants agreed on most questions regarding the management of patients suffering from depression and PD comorbidities. Opinions were based on their own clinical experience and available scientific evidence, and key consensus messages are wrapped up in Figure 2. In the following lines, consensus and controversial topics related to pathophysiological mechanisms of depression in PD, clinical parameters, and connections with other nonmotor symptoms as well as treatment options are addressed in depth in separate sections. This information can be used as guidance for other physicians given that diagnosis of depression in the context of PD is generally challenging and there are no specific guidelines available. Refining diagnosis is also important to reduce heterogeneity among trials and improve consistency. Treatment recommendations including newly commercialized antidepressants complement those found in general CPGs (either PD or depression-dedicated guidelines) from across countries as well as several published systematic literature reviews (SLRs) and meta-analyses.

### 4.1. Section I: Aetiology and Risk Factors for Depression in PD Patients

Previous evidence has shown that some neuropsychiatric disorders such as depression or anxiety can occur prior to PD diagnosis, sometimes even years before this is established [15]. In this regard, more than half of the panellists acknowledged that depression is a nonmotor and premotor symptom of PD (Table 1; S1). Moreover, there was major consensus on agreeing that depression is more frequent in future PD patients. Hence, most of responders were certain that depression contributes to the development of the disease, i.e., it can be considered a risk factor (Table 1; S2). In this regard, a recent meta-analysis of case-control and cohort studies performed by Wang et al. found a significant association between depression and an increased risk of subsequent PD [16].

On the contrary, the experts declared that depression would not qualify as a PD progression risk factor while responding to the diagnosis part of the questionnaire (Table 3; S21) and that motor and depression symptom intensities are not related with each other (Table 2; S17). In line with this question, consensus could not be reached when participants were asked about a putative relation between depression and distinct PD motor phenotypes (Table 1; S4). However, it seemed clear to most of participants that the presence of depression in the context of PD is indeed associated with greater severity of motor symptoms (Table 1; S5). Until now, some recent studies on emotional disorders and gait impairments suggest that depressive symptoms are worse [17, 18] and more frequent [19] during off periods, although they may not meet strict depressive disorder diagnostic criteria [20]. If mood and motor fluctuations are concomitant events [7], PD modifying agents such as dopamine agonists could delay or prevent psychiatric symptoms (Section IV: Treatment of Depression in PD Patients) [7]. Certainly, further research is warranted to unravel whether depression and motor symptoms are truly interrelated in PD patients and the nature of this link.

What held up to a vast majority of participants was that depression is associated with a higher prevalence of other nonmotor PD symptoms (Table 1; S7) as well as poorer cognitive performance (Table 2; S15). Again, literature shows some discrepancy with regards to the directionality of the latter association [21, 22]. A 4-year longitudinal study from Petkus and colleagues could detect that cognitive decline correlates with a subsequent increase in symptoms of anxiety and depression [21]. On the contrary, Wu et al. suggest that symptoms of depression that occur after PD onset are prodromal signs of dementia [22].

The pathophysiological link between depression and PD remains elusive. It is thought that a multifactorial model could explain why PD patients also suffer from depression, and this includes genetic susceptibility, adverse vital events and levels of support, coping strategies, and specific disease and psychosocial factors such as low educational and financial status [5, 20, 23]. When asked about specific risk factors, consensus was reached on female gender and older aged PD patients being associated with higher depression rates (Table 1; S6).

From a structural point of view, depressive patients share several brain alterations with PD patients alongside dopamine (DA), norepinephrine (NE), and serotonin depletion [24]. In the study presented herein, consensus was reached on the contribution of the following phenomena to the development of depression: neurotransmitter depletion (DA, serotonin, and acetylcholine), the impaired basal ganglia limbic system, and connections with orbitofrontal cortex (Table 1; S3 and S9). During early premotor stages of PD, experts acknowledged the existence of data pointing towards the impairment of the habenula having a role on depression (Table 1; S10). For instance, Borgonovo and colleagues suggested that the pathological hyperactivation of the lateral habenula may be at least partially responsible for affective disorders in PD through inhibition of dopaminergic and serotonergic neuronal activities [25].

### 4.2. Section II: Clinical Presentation of Depression in PD Patients

Depression in PD is a relevant matter from an epidemiologic point of view, the prevalence of which can reach high values but also vary significantly across studies depending on the setting (outpatient and inpatient clinics, nursing homes, and general population) and the diagnostic approach used [3]. In their systematic review, Reijnders et al. calculated a weighted prevalence for major depression and clinically relevant depressive symptoms of 17% and 35%, respectively [3]. Accordingly, Delphi participants agreed that up to one-third of PD patients can present with these symptoms (Table 2; S11).

There are other neuropsychiatric symptoms that PD patients may present with such as anxiety, apathy, and anhedonia [19]. Although very common (between 3.6% and 40% of PD patients), anxiety is another psychiatric comorbidity in PD patients that lacks robust recommendations to guide its diagnosis and management [7]. According to the expert panel, anxiety was considered a prevalent symptom in PD but not more than depression (Table 2; S13). Still, routine screening was deemed necessary for the majority of them, both at the start of the treatment and upon regimen change as symptoms of anxiety are thought to be enhanced by motor fluctuations [19] and anti-Parkinsonian drugs (Table 2; S14).

Unlike anxiety, apathy has been wrongly considered a clinical criterion of depression exclusively, but for more than a decade, efforts have been made to recognize it as a standalone syndrome too in older patients with at least some neurological impairment [26, 27]. In fact, international consensus was achieved in 2009 (and revised in 2018) to establish the diagnostic criteria and assessment tools for apathy, defined as the quantitative reduction of goal-directed activity in several dimensions (cognitive, emotional, or social) [27]. Thus, most participants agreed that apathy and depression require differential diagnosis (Table 2; S16). On the one hand, serotonergic affective symptoms such as hopelessness, sad mood, or suicidal thoughts would classify as key depression diagnostic criteria as they are not distinctive features of apathy, which has a nonserotonergic origin (Table 2; S18 and S19). Similar to apathy, experts agreed that anhedonia may be a consequence of the neurodegenerative process and unrelated to a depressive disorder (Table 3; S24).

On the other hand, the mere absence of the former affective symptoms would not rule out a diagnosis of depression. In these cases, it has been proposed that a key discriminant factor would be the low to absent subjective suffering in apathy compared with depression [20]. Additionally, experts have observed that depression can manifest not only as sadness but also as dysphoria (irritation) (Table 2; S20).

### 4.3. Section III: Diagnosis of Depression in PD Patients

Screening for depression in PD population was found required by the survey responders (Table 2; S12), and it coincides with the European Federation of Neurological Societies and Movement Disorder Society-European Section (EFNS-MDS-ES) and the Scottish Intercollegiate Guidelines Network (SIGN) recommendations [28–30]. According to the National Institute for Health and Clinical Evidence (NICE) and the Canadian Neurological Sciences Federation (CNSF) CPGs, clinicians should lower the threshold for diagnosing depression in these patients, especially for mild depression, since several clinical features of depression overlap with PD motor symptoms such as (hypophonic speech, lack of facial expression, sleep disorders, and reduced appetite) [29–33]. On top of that, gender may modulate the intricate repertoire of symptoms of depression discussed in Section II, although consensus on this topic could not be reached among participants (Table 3; S23). Hence, it seems reasonable to propose more studies to dissect clinical variables according to gender since prevalence of depression is not equivalent between women and men, as discussed earlier on (Table 1; S6).

Whether the use of depression scales in PD is essential remains an open question. Both SIGN and EFNS/MDS-ES CPGs recommend several validated tools such as the 15-item Geriatric Depression Scale (GDS-15), the Beck Depression Inventory (BDI), and the Hospital Anxiety and Depression Scale (HADS) [28–30], as long as diagnosis is not solely based on them but together with a clinical interview [29]. Of them, GDS-15 is the most specific screening tool for depression in PD, and together with HADS, they are not influenced by somatic symptoms, which makes them suitable for patients with notorious physical impairments [34]. In line with CPG recommendations, participants of this Delphi do not necessarily require tools to unequivocally diagnose depression in PD (Table 3; S22). While the opinion was robust among psychiatrists who found that patient interviews suffice the purpose, a subgroup of neurologists objected to it (data not shown). This observation may be interpreted as a need to provide more advanced training in psychiatric diagnosis to fellow neurologists. Regardless of the diagnostic method, there was a strong stand for the early treatment of depression symptoms in every PD patient, even in the absence of major depression (Table 3; S25).

### 4.4. Section IV: Treatment of Depression in PD Patients

The benefits of depression treatment are still unclear with the current available data [7]. A couple of meta-analysis of randomized clinical trials (RCT) by Bomasang-Layno et al. and Mills et al. found that pharmacological therapy of depression in PD ameliorates this condition [35, 36], while Troeung and colleagues determined a modest yet not a statistically significant effect of antidepressants compared with placebo [37]. Other more sceptical authors argue that antidepressants ensure only partial success (as in depression not associated with PD) and that poor adherence to treatment is associated to an increase in all-cause mortality in PD patients [38]. Overall, there is a need of comparative RCT to consistently pinpoint treatment efficacy and select one drug over another [7, 24, 39].

In order to establish consensus, experts were queried on a broad range of treatment approaches for depression in PD. About tricyclic antidepressants (TCAs), there are some evidence for treatment with amitriptyline, nortriptyline, and desipramine in PD [6, 7, 11, 40–43]. However, according to the literature and the expert panel opinion, their prescription within the scope of PD is questionable due to their anticholinergic adverse effects such as (cognitive impairment, orthostatic hypotension, increased risk of falls, constipation, and urinary retention) (Table 4; S26) [5, 29, 30, 40, 44]. Furthermore, other sources including the STOPP-START geriatric criteria distrust the administration of nortriptyline and amitriptyline in older PD patients [42, 45]. Bearing that in mind and compared with other TCAs, nortriptyline may be the safest option given its lower risk of anticholinergic effects and therapeutic window [5]. As such, it was also deemed the safest option among tricyclic antidepressants in PD (Table 4; S29). Nevertheless, when tolerability was taken into account together with efficacy, consensus was not achieved (Table 4; S46).

Among selective serotonin reuptake inhibitors (SSRIs), sertraline was voted as the safest drug among its class (Table 4; S27), which was considered a good treatment approach to tackle depression in the context of PD (Table 4; S42). SSRIs are also recommended by the American Psychiatric Association (APA) CPG as well as the NICE, the Spanish NHS, and the EFNS/MDS-ES guidelines, but they do alert on the potential exacerbation of PD symptoms associated to SSRI uptake [31, 40, 44, 46]. Here, consensus could not be reached when discussing the deleterious effect of both SSRIs and dual-action serotonin and norepinephrine reuptake inhibitors (SNRIs) on motor symptoms (Table 4; S30), which may be linked to the existence of controversial results in previous studies. On the one hand, recent observational studies have documented an association between the prescription of some SSRIs, SNRIs, and other antidepressants and the incidence of movement disorders [47–49]. On the other hand, former RCTs had not observed significative effects on the motor function when comparing SSRIs (paroxetine and escitalopram) with dual SNRIs (venlafaxine and duloxetine) in PD patients [50, 51]. Regarding other side effects, they seem to increase the risk of pharmacological interactions (fluoxetine, paroxetine, and fluvoxamine) and dose-dependent cardiac arrythmia (citalopram and escitalopram), although the FDA and Health Canada-dose limitations in older patients have been challenged by recent data [52].

Given their efficacy and tolerability, the multimodal antidepressant vortioxetine and the dual-action SNRIs venlafaxine, desvenlafaxine, and duloxetine were considered good options for PD patients with depression (Table 4; S28, S40, S43). Starkstein et al.'s review on RCT, patient-control studies, and case series concluded that both SSRIs and SNRIs are the gold standard treatments, with TCAs being used when patients do not respond well to the second SSRI or SNRI [5]. Last year, the International Parkinson Disease and Movement Disorder Society (IPMDS) identified some SSRIs (sertraline, citalopram, paroxetine, and fluoxetine) and a SNRI (venlafaxine) as possibly or clinically useful for depression in PD [6]. Other consensus recommendations on the administration of SNRIs in the elderly [11, 53, 54] and in PD patients are found elsewhere [42, 44]. There are no studies assessing the benefits and safety profile of desvenlafaxine or the newly commercialized vortioxetine in depression and PD comorbidities, yet a very recent postmarketing study with a WHO pharmacovigilance database has documented some harmful associations between movement disorders and a plethora of old and new generation antidepressants.

Following efficacy and tolerability criteria (especially regarding the absence or a minimal increase in motor symptoms), other supported drugs for PD and depression comorbidities were bupropion (NE-DA reuptake inhibitor; Table 4; S33 and S45), mirtazapine (NEergic and specific serotonergic antidepressant; Table 4; S31 and S44), and tianeptine (glutamatergic modulator; Table 4; S35 and S48). Bupropion may be an ideal candidate given its dopaminergic action, lack of serotonergic activity, and subsequent low risk of Parkinsonism but may also induce psychotic symptoms [11, 42, 44]. A part from the revision from Costa and colleagues, no other specific studies addressing mirtazapine in PD patients have been found, but its extended use in psychogeriatric population is well known, which may be due to a higher antidepressant response of multitarget drugs such as mirtazapine and dual-action agents in neurodegenerative conditions [42, 54]. In spite of its relatively short marketing time, all participants agreed with the efficacy of tianeptine in depressed PD patients (Table 4; S35). This is accompanied by the previously observed decrease in Hamilton and BDI depression scales scores in the subgroup of PD patients treated with this drug, particularly in those with mild depressive, motor, and cognitive symptoms [55].

No conclusions could be drawn on whether agomelatine and trazodone constitute good treatment choices in this context (Table 4; S32, S37, S47 and S49). There is still little evidence on both drugs, but it is thought that agomelatine may bear double therapeutic potential as a safe antidepressant and a circadian rhythm regulator due to its novel melatonergic mechanism of action [56, 57].

There is also weak evidence supporting the role of anti-Parkinsonian drugs in depression treatment [7], although APA, EFNS/MDS-ES, and CNSF guidelines recommend them with substantial confidence [32, 44, 46]. The expert panel seconded the use of dopamine agonists (pramipexole, ropinirole, and rotigotine) (Table 4; S34) as several controlled studies and meta-analyses have proven them effective in this scenario [5, 6, 58–60]. However, caution should be taken when prescribing them to PD patients. A recent study that was published after concluding the present Delphi survey showed that depression predisposes to the development of impulse control disorders (ICDs) in these patients and that this risk is magnified by dopamine agonists [61]. Also, they should be replaced by other pharmacological options such as antidepressants when patients do not respond to 3 mg and 15 mg daily doses of pramipexole and ropinirole, respectively [59]. Caution should also be taken when combining serotonergic medications with type B monoamine oxidase inhibitors (MAO-BIs) such as selegiline to avoid serotonin syndrome (Table 4; S38) [31, 44, 62]. Unlike serotonin-enhancing antidepressants, tianeptine was considered safe together with Parkinson's medication (Table 4; S39). Thus, personalized management of depression in PD is key to avoid drug interactions and side effects due to any coexisting therapies [31, 32, 40].

Main CPGs do not discard nonpharmacological therapies, but they specifically state that there is insufficient evidence to formally recommend them in patients with PD and depression [31, 32, 40, 44, 46]. Nonetheless, there is considerable amount of reported experience on electroconvulsive therapy (ECT) by psychiatrists and, to a lesser extent, neurologists and geriatrists (data not shown). ECT is specifically used in patients with drug-refractory and life-threatening affective disorders (Table 4; S41) [5, 11, 63].

Cognitive behavioural therapy (CBT) is mostly indicated when symptoms of depression are mild [5]. A controlled RCT assessing the efficacy of CBT versus clinical management alone in PD patients observed significant improvements in cognitive and behavioural (but not somatic) symptoms of depression [64], which may have required antidepressant prescription. Consensus on its appropriateness in PD patients was achieved in this study (Table 4; S36), and it may well be considered when antidepressants are contraindicated or undesired [5].

## 5. Conclusions

This Delphi-based study provides an expert opinion on the aetiology and clinical manifestations of depression in PD and offers specific diagnostic and treatment hints (Figure 2). In summary, the presence of depressive symptoms was considered a PD risk factor, but it should not be used as a surrogate of motor symptom exacerbation during the progression of the disease. Female gender, older age, and brain structural factors together with neurotransmitter depletion could contribute to the development of depression in the context of PD. Thus, PD patients should be systematically screened for depression and anxiety, the diagnostic of which can be aided using clinical scales such as the GDS. Anhedonia and apathy should be ruled out as they are not necessarily linked to depression. The latter is characterized by the presence of affective symptoms of hopelessness, sadness, and suicidal thoughts or dysphoria.

Regardless of the clinical severity, symptoms of depression require treatment. SSRIs, SNRIs, vortioxetine, bupropion, mirtazapine, tianeptine, and dopamine agonists were considered efficacious and well-tolerated pharmacological options for depression in PD. TCAs and combining MAO-BIs with serotonergic antidepressants should be of limited use in this context due to significant side effects. ECT is especially indicated for severe and drug-refractory cases, whereas CBT constitutes a nonpharmacological option worth exploring in patients with mild depression. Future large-scale studies are needed to consolidate this information and recommendations for depression in PD.

## Figures and Tables

**Table 1:** Section I: aetiology and risk factors for depression in PD patients.

Statement		Consensus agreement	Consensus disagreement	No consensus
S1	Depression is a nonmotor and premotor symptom of PD.	✓		

S2	Depression is more frequent in people who will later suffer from PD; thereby, it can be considered a risk factor.	✓		

S3	Decreased levels of DA, serotonin, and acetylcholine are depression trigger factors in PD.	✓		

S4	Depression in PD is not more frequently related to any motor phenotype.			-

S5	Depression in PD is associated with more severe motor symptoms.	✓		

S6	Female gender and older age correlate with an increased risk of PD-related depression.	✓		

S7	Depression in PD is associated with a higher prevalence of nonmotor symptoms.	✓		

S8	Having suffered from cancer and not being married correlate with an increased risk of PD-related depression.			-

S9	Impaired basal ganglia limbic system and connections with the orbitofrontal cortex are involved in the pathophysiology of PD-related depression.	✓		

S10	The habenula is an amine brain integrative area, and there are data supporting that its impaired function could contribute to depression in early premotor PD stages.	✓		

PD, Parkinson's disease; DA, dopamine.

**Table 2:** Section II: clinical presentation of depression in PD patients.

Statement		Consensus agreement	Consensus disagreement	No consensus
S11	Up to one-third of PD patients have clinically relevant symptoms of depression.	✓		
S12	Systematic assessment of PD-related depression is needed due to its high prevalence.	✓		
S13	Anxiety is a very frequent symptom in PD.	✓		
S14	Anxiety may be related to treatment of PD.	✓		
S15	Depression in PD increases the risk of cognitive symptoms.	✓		
S16	In PD, apathy and depression require differential diagnosis.	✓		
S17	In patients with depression and PD comorbidities, depressive signs and symptoms and motor deficit intensities are not correlated.	✓		
S18	Symptoms such as hopelessness, sad mood, or suicidal thoughts are keys for the diagnosis of PD-related depression.	✓		
S19	Apathy in PD is not characterized by hopelessness, sad mood, or suicidal thoughts.	✓		
S20	Patients with depression and PD comorbidities can present both dysphoric (irritable) and sad mood.	✓		

PD, Parkinson's disease.

**Table 3:** Section III: diagnosis of depression in PD patients.

Statement		Consensus agreement	Consensus disagreement	No consensus
S21	Depression is a PD progression symptom.		✗	
S22	Depression scales such as Beck, Hamilton, Montgomery, GDS, or UPDRS scales are needed to identify depressive symptoms in PD patients.		✗	
S23	Clinical features of depression in PD differ between men and women.			-
S24	Anhedonia can be observed in PD patients, and it may not be associated with depressive symptoms.	✓		
S25	Symptoms of depression in PD patients do not require treatment except in the presence of a major depressive disorder diagnosis.		✗	

PD, Parkinson's disease; GDS, Geriatric Depression Scale; UPDRS, Unified Parkinson's Disease Rating Scale.

**Table 4:** Section IV: treatment of depression in PD patients.

Statement		Consensus agreement	Consensus disagreement	No consensus
S26	TCAs have important side effects in PD patients.	✓		
S27	The best-tolerated SSRI for PD patients is sertraline.	✓		
S28	The dual SNRIs are effective in PD patients with major depression.	✓		
S29	Among TCAs, nortriptyline shows the best safety profile in PD patients.	✓		
S30	SSRIs and SNRIs (dual) worsen motor symptoms of PD.			-
S31	Mirtazapine is an effective and well-tolerated antidepressant drug for PD patients.	✓		
S32	Agomelatine, with norepinephrinergic and indirect dopaminergic effects, is not a drug of choice in PD patients.			-
S33	The DA and NE reuptake inhibitor bupropion is effective in PD patients.	✓		
S34	Nonergotic DA-receptor agonists (ropinirole, pramipexole, and rotigotine) are effective for treating PD-related depression.	✓		
S35	The glutamatergic modulator tianeptine is effective for depression in PD patients.	✓		
S36	CBT is effective to treat depression in PD patients.	✓		
S37	Trazodone is an effective treatment for depression in PD.			-
S38	Combining anti-Parkinsonian MAO-BIs and serotonergic antidepressants is of limited use given the possible occurrence of a serotonin syndrome in PD.	✓		
S39	Tianeptine is a safe antidepressant in depressive PD patients under treatment with anti-Parkinsonian drugs.	✓		
S40	Vortioxetine is a multimodal antidepressant that is well tolerated in PD patients.	✓		
S41	ECT is an advisable therapeutic approach in patients with dementia and depression that show poor response to antidepressants.	✓		
S42	Given their PD-specific efficacy and tolerability, SSRIs are a good treatment option for depression in PD patients.	✓		
S43	Given their PD-specific efficacy and tolerability, dual and multimodal drugs are a good treatment option for depression in PD patients.	✓		
S44	Given its PD-specific efficacy and tolerability, mirtazapine is a good treatment option for depression in PD patients.	✓		
S45	Given its PD-specific efficacy and tolerability, bupropion is a good treatment option for depression in PD patients.	✓		
S46	Given its PD-specific efficacy and tolerability, nortriptyline is a good treatment option for depression in PD patients.			-
S47	Given its PD-specific efficacy and tolerability, agomelatine is a good treatment option for depression in PD patients.			-
S48	Given its PD-specific efficacy and tolerability, tianeptine is a good treatment option for depression in PD patients.	✓		
S49	Given its PD-specific efficacy and tolerability, trazodone is a good treatment option for depression in PD patients.			-

TCAs, tricyclic antidepressants; PD, Parkinson's disease; SSRIs, selective serotonin reuptake inhibitors; DA, dopamine; NE, norepinephrine; SNRIs, serotonin and NE reuptake inhibitors; MAO-BIs, type B monoamine oxidase inhibitors; ECT, electroconvulsive therapy; CBT, cognitive behavioural therapy.

## Data Availability

The datasets used to support the findings of this study are available from the corresponding author upon request.
